# High-School Satisfaction Scale (H-Sat Scale): Evaluation of Contextual Satisfaction in Relation to High-School Students’ Life Satisfaction

**DOI:** 10.3390/bs9120125

**Published:** 2019-11-23

**Authors:** Ernesto Lodi, Diego Boerchi, Paola Magnano, Patrizia Patrizi

**Affiliations:** 1 Department of Humanities and Social Sciences, University of Sassari, 07100 Sassari, Italy; patrizi@uniss.it; 2 Department of Psychology, Università Cattolica del Sacro Cuore, 20123 Milan, Italy; diego.boerchi@unicatt.it; 3 Faculty of Human and Social Sciences, Kore University, 94100 Enna, Italy; paola.magnano@unikore.it

**Keywords:** high school satisfaction, adolescent’s well-being, life satisfaction

## Abstract

Recent literature on positive psychology underlines the crucial role of schools to create a psychologically healthy environment and to set programs and strategies fostering adolescents’ well-being. The aim of the present study is to validate a scale that measures scholastic satisfaction since a scientific evaluation and interventions on school satisfaction can help professionals to support adolescents’ positive development and school adjustment. We adapted the College Satisfaction Scale (CSS) and confirmed the previous five-dimensional structure also in a high school students’ sample (n = 792). The High-school Satisfaction Scale (H-Sat Scale) evaluates five dimensions of school satisfaction: appropriateness of choice (CH), quality of school services (SE), relationships with classmates (RE), effectiveness of study habits (ST) and usefulness for a future career (CA). The questionnaire consists of 20 items; it showed good psychometric features and, consistent with previous literature, confirmed its validity in relation to life satisfaction and quality of life of high school students. Compared with previous scales, the H-Sat evaluates two innovative areas of school satisfaction since it gives a measure of satisfaction in career path (appropriateness of choice and usefulness for future career) could help school counsellors to set interventions in this field.

## 1. Introduction

Research and studies on adolescents’ health have often focused on negative behaviours related to adverse health outcomes, but the most recent literature is looking mainly at protective factors [1]. In the last decades many studies, adopting perspectives related on improving the level of quality of life, have focused on the detection of factors promoting people well-being. It is both an individual and a social level issue, since understanding adolescents’ needs related to mental health is a basilar issue to let young people not only to fulfil their potential but also to contribute to the development of our communities [2]. Various aspects have stimulated the study of adolescents’ well-being. For example, in an educational context, the evaluation and the professionals’ intervention on students’ satisfaction is a crucial way to avoid dropout phenomenon and all the factors associated with students’ disengagement. Indeed, satisfaction with the school and other domain-specific satisfaction in educational context are related to academic performance, engagement and academic progress at all educational levels, from primary school to the college [3,4,5,6]. Wilkins and colleagues [7] underlined that domain-specific satisfaction predicted sub-dimensions of intended academic persistence and Cock and Halvari [8] highlighted that motivation to achieve success is positively correlated with school performance and school satisfaction. Another source of scientific interest in this field comes from positive psychology, since the positive view of youth development contrasts with traditional approaches focused on problems that young people may face while growing up [9]. Positive perspectives try, at a school level (1) to understand how implement positive education; (2) to set mental health and well-being strategies; (3) to design programs that can have an impact on students and school-staff well-being. According to Baker et al. schools can influence students’ positive adjustment, and they have to work on building a psychologically healthy environment to support people’s development in this context [10]. Then, Kern et al., given the benefits of positive education, highlighted that “schools need to consider how to build best and support students’ well-being” ([11], p. 262). Lopez and colleagues, analysing the variability of protective emotional dimensions in the lives of people, underlined the crucial role of family, school and community to improve the happiness and well-being of children, young and adults, also in a multicultural context [12]. Promoting positive adjustment and create a healthy environment at school is a basilar approach to promote the adolescents’ development since research in adolescence showed that life, school and relationship satisfaction is a significant predictor of positive outcomes in a variety of life domains [13]. The positive outcomes can be identified for example in improved emotional, social, physical and behavioural health, better social relationships, academic engagement and achievement [14,15,16,17,18]. 

Huebner and Gilman [4], highlighting a scarcity of research on school satisfaction during recent years, affirmed that “school-related affective variables, such as school satisfaction have received little research attention, even though such variables may relate directly and/or indirectly to academic success” (p. 140). Moreover, research that has studied adolescents’ well-being is more concentrated on the influences of family and individual characteristics, not considering in depth the amount of time spent by the adolescents in school and the influence of this context on adolescents’ identity, roles and feelings [19,20,21]. For this reason, there is a crescent demand for reliably and standardised instruments to evaluate domain-specific satisfaction at school. On this theme, we intend to present the adaptation of a scale for high school students (High-school Satisfaction Scale; H-Sat Scale) already validated in the version for university students: The College Satisfaction Scale (CSS) [3]. The dimensions of the scales have been identified starting from the analysis of the scientific literature and the tools used in this field. The structure of the scale covers five areas, that were shown to affect domain-specific satisfaction, in previous literature: appropriateness of the students’ university choice, quality of the university services, relationships with his/her colleagues, quality of his/her study habits and usefulness of course degree attended for his/her future career. The innovative value of the scale is the possibility to measure not only some dimensions related to traditional view of school satisfaction (relationships, study habits, school environment) but also if the persons feel satisfied with their career goals and career projects because of the school attended in terms of programs, knowledge and competencies acquired. For this reason, the adaptation of this instrument can be useful for school staff and professionals of career guidance such as school counsellor, helping students to work on their positive school adjustment, on their career plans, on the improvement of some areas of school well-being.

## 2. Literature Review

### 2.1. Evaluation of School Satisfaction and Related Variables

School satisfaction can be defined as a cognitive-affective evaluation of overall satisfaction with school life experience [19], and is related to many kinds of variables in the scientific literature on this topic. In fact, many studies underline that it is a complex multidimensional construct affected by a series of individual, behavioural, relational and environmental factors. Konu and Rimpela, after a review of psychological, sociological, and educational literature, described their “School Well-being Model” as composed by four dimensions: school conditions (having), social relationships (loving), means for self-fulfilment (being) and health status [22]. A review conducted by Upadyaya and Salmela-Aro showed that school engagement is negatively associated with students’ ill-being, fostering positive emotions, life satisfaction and academic success [23]. Students with low school well-being more likely tend to show biased attention towards words describing school-related stressors [24]. Many studies, analysing the relations between positive psychology variables and school satisfaction, found that school well-being and satisfaction was predicted by positive constructs such as gratitude, hope, optimism, courage and career adaptability [25,26,27,28]. Other individual dimensions affecting academic satisfaction can be recognised in school self-efficacy and school grit [6,29,30,31,32].

At relational and environmental levels, several variables demonstrated to affect school satisfaction and general well-being of adolescents. Indeed, having good relationships can diminish psychosocial stress [2] and improve subjective well-being since it is strongly related to the availability of social resources [33]. The positive school environment can also avoid risky behaviours since perceived parent-family and school connectedness are protective factors to prevent health risky behaviours [34,35]. The influence of social and environmental variables on school satisfaction was reported in numerous studies: supportive teacher behaviour, positive peer relations and the ability to involve parents about their school experiences, school climate, support from parents, teachers and classmates [19,36,37,38,39,40]. Zullig, Huebner and Patton [39] have examined environmental factors related to adolescents’ satisfaction and school experience, revealing that the factors associated to school climate (e.g., social support, relationship with teachers) are crucial correlates of school satisfaction, and their effect was no sensitive to demographic variables and academic performance levels. Positive interpersonal relationships at school, such as receiving support from peers and teachers, led to higher levels of general life satisfaction and happiness [41,42]. Ito and Smith [43] indicated interpersonal support as the best predictor of school satisfaction and Lester and Cross underlined that in the second year of secondary school peer support was the most potent protective factor for mental well-being [44]. In a recent study, Lemma and colleagues found that academic stress in 15-year-old adolescents is associated with lower physical and psychological well-being and with lower quality of relationships with parents and peers [1]. The authors theorised that school support environment could represent the main factor affecting subjective well-being. In another study, Tian, Liu, Huang, and Huebner [45] found differences about different group age in adolescence: during early adolescents, only parents and teachers support (not friends) was related to positive school well-being, on the other hand during middle adolescence, only friends and teachers support (but not parents support) was related to school well-being. Wong and Siou demonstrated that two dimensions of school climate (school order and environment, perceived privileges) predicted the level of students’ happiness, while the most related variable to the school satisfaction was to feel competent at school [19]. The role of particular aspects of educational environment (f.e. services for students such as appropriateness of library, adequate materials, number of classrooms) on academic satisfaction and academic progress was studied both in schools and university context [46,47,48,49]. Specifically, in the Konu and Rimpela model, the dimension of *having* concerns the school condition (safety and protective environment, size of classes, adequate services such as health services and counselling services, possibility to receive assistance) that can facilitate the educational activities [22]. Savolainen and colleagues found that school organisation and physical working environment affected pupil’s well-being [50]. Graham and Gisi underlined that the educational environment encourages students to follow educational activities in the classrooms and several studies indicated that adequacy of school resources was also associated with parents’ school satisfaction [51,52].

Finally, many studies at school or college level have shown the close relationship between academic competences (or feel competent), academic performance and academic satisfaction [19,53,54,55,56]. For example, Huebner and Gilman demonstrated that very high level of school satisfaction was significantly associated with global life satisfaction, hope, internal locus of control, and GPA [4]. Moreover, in a total sample of 341 secondary school students’, only three students in the group of very high school satisfaction and nine students with average level of satisfaction reported clinical levels of psychological symptoms.

### 2.2. The Relation between Domain-Specific Satisfaction and Overall Life Satisfaction

Adolescents’ well-being cannot be considered a one-dimensional construct, but it refers to multiple and various domains: self, relations with family and friends, environment and school [21]. Research underlined that school satisfaction has a crucial role in students’ quality of life [21,22,23,24,25,26,27,28,29,30,31,32,33,34,35,36,37,38,39,40,41,42,43,44,45,46,47,48,49,50,51,52,53,54,55,56,57]. A central construct to evaluate global satisfaction of adolescents is life satisfaction, that only recently has received proper attention in adolescence, as more attention has been paid to it regarding other age groups, such as adults [58,59]. Several authors theorise subjective well-being among adolescents as composed by three-components: overall life satisfaction, positive affect and negative affect [60,61]. Life satisfaction is a component of subjective well-being and can be defined as a cognitive judgment of life in relation to a series of criteria personally defined [62]. Life satisfaction has two main effects: (1) promotes positive resources of the individuals since is linked to many positive outcomes in various spheres of life; (2) it is a preventive factor in facing adversities and stressful events in own life, as shown by a longitudinal study [59,60,61,62,63]. The relationship between domain-specific and overall satisfaction is not yet clear to define. Gilman and Huebner theorise school satisfaction as a part of global students’ life satisfaction, corroborating a vision of domain satisfaction in a valued context as a relationship between the part and the whole represented by overall life satisfaction [64,65]. As Funk, Huebner and Valois underlined, since there are many studies that emphasize the relationship between life satisfaction and numerous dimensions (community and economic conditions, family and peer relationship, school experience) that life satisfaction measures, it could be used “as part of comprehensive assessments of adolescent health” ([66], p. 42).

Several authors demonstrated the relationships between domain-specific and general life satisfaction, observing the two constructs affecting each other, like a sort of *osmosis* process [67,68]. Research suggest for both a bidirectional path, but, on the other hand, a longitudinal study found that the path from life satisfaction to domain-specific satisfaction is stronger [69,70]. The link between domain-specific satisfaction and overall life satisfaction can also be conditioned by cultural variables: for example, Park and Huebner, among other results, demonstrated that only for Korean adolescents satisfaction with “school” contributed significantly to global life satisfaction, while for American adolescents it was satisfaction in the area of “Self” [71]. The authors explained this difference in terms of different culture frameworks (individualistic vs collectivistic). 

Finally, the direction from domain-specific satisfaction to life satisfaction seems stronger when the specific context is a particularly central life domain, related to individuals’ life and their identity [72]. We can consider school, such as familiar context, as the most crucial context affecting identity and the role of student as central between the various roles that a teenager assumes. The importance of roles and identity in adolescents’ well-being is supported from theories and research that underline specifics variables crucial for a positive development in this group age such as: management of new social relations, support of significant people (parents, teachers) and positive family relations, proper expectations of effectiveness, good sense of belonging in friendship and school relations, participation in public life and to leisure activities, possibility of developing one’s abilities, challenges related to the task of individuation and to the separation from the family, good decision making skills [73,74,75,76]. 

### 2.3. Assessment of School Satisfaction

After a review of the relevant literature, we have found that the most used scales on school satisfaction are the following:

Brief Adolescents’ Subjective *Well-Being in School* that gives a comprehensive measure of adolescents’ subjective well-being (SWB) at school and was developed from Adolescents’ Subjective Well-Being in School Scale (ASWBSS) [77,78].

*My Life as a Student* aims to assess the quality of life of students (15–19 years old) [73]. It is composed of seven scales related to the school experiences and life in general, evaluating satisfaction with school experience; opportunities to make decisions autonomously; relationships with classmates; current life conditions; relationships with family members; praise received when due; help availability. 

The Questionnaires for the evaluation of school well-being and identification of risk factors (QBS) analyses the well-being of children and teens (age 8–13) through three questionnaires from three points of view: parents, teachers and the pupil himself [79]. The version for teens evaluates the following dimensions: satisfaction and acknowledgement, relationship with teachers, relationship with classmates, emotional attitude at school, self-efficacy, causal attribution processes.

Konu et al. using confirmatory factor analysis on the data collected with School Health Promotion Survey, confirmed the four dimensions of the theoretical model of School Well-being [22,80]. The dimensions were: school conditions, social relationships, means for self-fulfilment and health status.

Van Landeghem designed a well-being questionnaire with eight indicators: well-being at school, social integration in the class, relationships with teachers, interest in learning tasks, motivation towards learning tasks, attitude to homework, attentiveness in the classroom and academic self-concept [81]. 

Sometimes satisfaction at school is a sub-dimension evaluated by more general questionnaire on well-being, for example in: (1) “The Multidimensional Students Life Satisfaction Scale”(MSLSS) designed by Huebner, with also a brief form (BMSLSS) published by Funk, Huebner and Valois, where school satisfaction contribute with other dimensions (such as Family, Friends, Living Environment and Self) to the students well-being; (2) as school engagement in the attempt of Kern, et al. to evaluate in students the dimension of Seligman PERMA model of psychological well-being (positive emotions, engagement, relationships, meaning, and accomplishment) [11,66,82].

The H-Sat Scale could be more useful to professionals involved in supporting the development of carrier projects because, differently from the previous ones, it detects, in addition to the classic indexes linked (for example quality of the relationships), also satisfaction areas related to the choice and the perceived utility of the schools for their career path. It is composed only by 20 items, but it can reflect the multidimensional nature of school well-being.

## 3. Pilot Study

The aim of this study was to adapt and test the College Satisfaction Scale (CSS) [3] on high-school students. The items were adapted after a discussion with a class of high-school students. A pilot study aimed to confirm the construct validity and the reliability of the scale. The primary study aimed to confirm its relationship with general life satisfaction and academic performance. The aim of the pilot study was to test the basic structure of the H-Sat Scale by:

(1) Testing the factorial structure through exploratory factorial analysis (EFA);
(2) Providing evidence regarding the internal consistency through Cronbach’s alfa.

### 3.1. Scale Adaptation

In the first step, each author adapted the scale autonomously, and, after a comparison, some items were modified. Following, the scale was submitted to a class of 21 high-school students with the aim of testing if all the items were understandable and with adequate variance in the answers. After the administration, a focus group provided some suggestions to modify some of the items.

### 3.2. Participants

A pilot study was conducted on 364 Italian high-school students very heterogeneous for the course attended and class. The sample was somewhat paired by gender (54 % females and 46 % males) whose ages ranged from 14 to 19 years old (*M* = 16.40; *SD* = 1.576).

### 3.3. Procedure

The questionnaire, composed of 20 items, was administered to the students in the paper-pencil version during the school time. They were asked to fill the questionnaire anonymously, indicating how satisfied they were with each sentence using a 5-point Likert scale: (1) ’not at all’, (2) ‘a little’, (3) ’somewhat’, (4) ‘very’, (5) ’completely’.

### 3.4. Results

Descriptive statistics showed the normal distribution for all the items with skewness and kurtosis ranging from −0.645 to 0.483, mean ranging from 2.38 to 3.70 and SD ranging from 0.945 to 1.166. We tested the scale structure through EFA using the maximum likelihood method, with oblimin with Kaiser normalisation rotation, and the IBM Corp. Released 2017. IBM SPSS Statistics for Windows, Version 25.0. Armonk, NY: IBM Corp. Considering both the original structure and the screen plot analysis, the five-sub-scales’ structure appeared to be the best solution, explaining 68.54% of the variance. As you can see in Table 1, the structure was well defined with just one item which partially saturated two factors. Finally, internal consistency of all the sub-scales was also good with Cronbach’s alpha values ranging from 0.818 to 0.926. The final version of H-Sat Scale, confirming structural dimensions of CSS Scale, asses 5 areas of school satisfaction: appropriateness of choice (CH), quality of school services (SE), relationships with classmates (RE), effectiveness of his/her study habits (ST), usefulness for future career (CA).

## 4. Main Study

The main study sought evidence of the validity and reliability of the H-Sat Scale by:

(1) Testing latent factorial structure through Confirmatory Factorial Analysis (CFA);
(2) Providing evidence regarding the internal consistency through Cronbach’s alpha;
(3) Testing its concurrent validity with a different questionnaire on student’s quality of life;
(4) Testing its concurrent validity with a different scale on general life satisfaction.
(5) Testing its concurrent validity with academic performance.

An additional aim of the study is to verify whether there are significant differences in the areas of satisfaction considering the course year attended by the students. Indeed, the scholastic familiarity, increasing the awareness of strengths and weaknesses of the school attended, could influence the different experiences of the school context.

### 4.1. Participants

The main study was conducted on 792 Italian high-school students attending three different types of school: linguistic lyceum, scientific lyceum, and technical institute. The samples were not equilibrated by gender: in linguistic lyceum, students were mainly females (84.2%), whereas males were more numerous in scientific lyceum (75.6%) and technical institute (95.9%). Instead, the three subsamples were homogeneous by age, ranging from 14 to 20 years old (*M* = 16.15; *SD* = 1.569).

### 4.2. Procedure

During school time, students divided by class were conducted in the computer classroom where one of the authors asked them to fill out an on-line questionnaire “about their experience as a high-school student”. The students were previously informed that the compilation was nominal and would provide a personal profile and that they were free to decide if participate or not to the study. The H-Sat Scale was administered before the My Life as a Student, the Satisfaction With Life Scale (SWLS) and some questions on demographic data (sex, age, course attended and class) [73,74,75,76,77,78,79,80,81,82,83,84,85,86].

### 4.3. Measures

H-Sat Scale. The questionnaire was the same used in the pilot study. Descriptive statistics showed the normal distribution for all the items for all the three sub-samples with skewness and kurtosis ranging from −0.680 to 0.487, mean ranging from 2.45 to 3.69 and SD ranging from 0.809 to 1.083. As you can see in Table 2, also scales’ scores were normally distributed. The scale is available in Appendix A.

My Life as a Student [73]. This is a questionnaire that aims to assess the quality of life of students from 15 to 19 years old on a 5-points Likert scale. The questionnaire is composed of seven scales: satisfaction with the school experience (seven items, α = 0.86); satisfaction with opportunities to make life decisions autonomously (five items, α = 0.68); satisfaction with relationships with classmates (three items, α = 0.67); satisfaction with current life conditions (three items, α= 0.78); satisfaction with relationships with family members (four items, α = 0.87); satisfaction with praise received when due (two items, α = 0.73); and satisfaction with help availability (two items, α = 0.81). Some scales refer to the school experiences, some to life in general.

Satisfaction With Life Scale — SWLS [86]. It is a one-dimensional scale on general satisfaction with life composed of five items. Respondents are asked to provide their agree on each sentence on a 7-point Likert scale (from ‘strongly disagree’ to ’strongly agree’). Di Fabio and Gori [87] confirmed the factor structure, reliability, concurrent validity and the psychometric properties of the Italian version of Satisfaction With Life Scale (SWLS) also in a sample of high school students and young adults. With this sample, all items were normally distributed; structural validity, tested with CFA, showed optimum goodness-of-fit-indexes (χ^2^ = 12.003, df = 5, *p* = 0.035; RMSEA = 0.042; CFI = 0.996); reliability was also good with Cronbach’s alpha equal to 0.834.

Academic performance. At a first step, we compared the unweighted sum the grades of all the subjects studied by the students, and the one weighted by the weekly number of hours of each subject: because the weighted one was less related with the H-Sat scales, only the data related to the unweighted one are here considered.

### 4.4. Results

**Figure 1:**
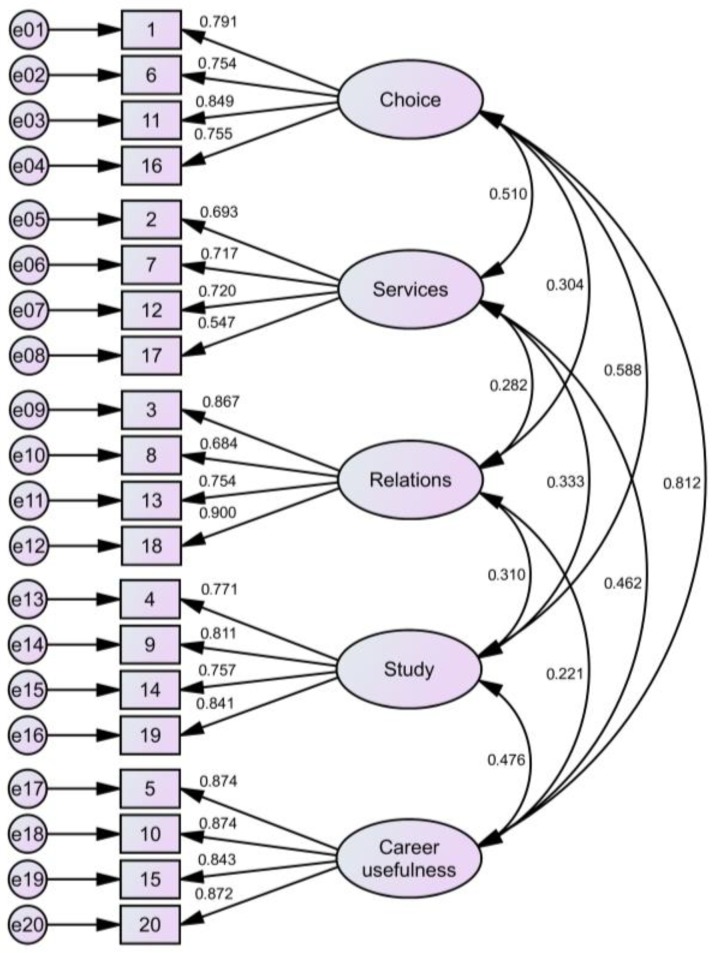
Construct validity: H-Sat CFA with standardised estimates.

H-Sat Scale Construct Validity. We tested the scale structure through CFA using the maximum likelihood method and the IBM Corp. Released 2017. IBM AMOS for Windows, Version 25.0. Armonk, NY: IBM Corp. Goodness-of-fit indexes were examined through the chi-square test, root mean square error of approximation (RMSEA) and comparative fit index (CFI). Even if a non-significant chi-square is desired, which would suggest that the observed and reproduced covariance matrix does not significantly differ, models with a large sample can only be evaluated by RMSEA and CFI because this test is sensitive to sample size [83]. Models with acceptable fit also presented RMSEA < 0.08 and CFI > 0.90, whereas models with optimum fit presented RMSEA < 0.05 and CFI > 0.95 [84,85]. Structural validity, tested with CFA, showed acceptable goodness-of-fit-indexes (χ^2^ = 790.589, df = 160, *p* < 0.000; RMSEA = 0.071; CFI = 0.934), and standardized regression weights ranging from 0.547 to 0.900. All the scales were significantly related with correlations ranging from 0.221 to 0.812 (see Figure 1 for details).

H-Sat Scale Reliability. Internal consistency was assessed for the five subscales, and all Cronbach’s alpha indexes were optimum for the whole sample and the three sub-samples (see Table 3 for details).

H-Sat Scale Concurrent Validity with My Life as a Student Scale. H-Sat Scale scores were compared with those of My Life as a Student Scale. We expected several positive and significative correlations, mostly with satisfaction with the school experience for all H-Sat subscales, satisfaction with relationships with classmates for H-Sat relationships, and satisfaction with praise received when due for H-Sat study. Correlations between the three subsamples were fairly similar, so we decided to consider just those of the whole sample. Table 4 shows that all the correlations were positive and statistically significant and that the strongest ones confirmed our hypothesis.

H-Sat Scale Concurrent Validity with SWLS. We hypothesised that high-school satisfaction is related to general life satisfaction. As showed in Table 5, all the scales are significantly related to general life satisfaction, and study and choice are the two most related.

H-Sat Scale Concurrent Validity with School Performance. We hypothesised that high-school satisfaction is partially related to school performance. As shown in Table 5, study, followed by choice and career utility, was significantly related to school performance.

We tested the variability of the contextual satisfaction according to the class attended with ANOVA and Scheffe posthoc test, a very conservative method to correct alpha for multiple mean comparisons. The differences between the five classes attended were statistically significative for career usefulness, choice, and services (Table 6).

**Figure 2:**
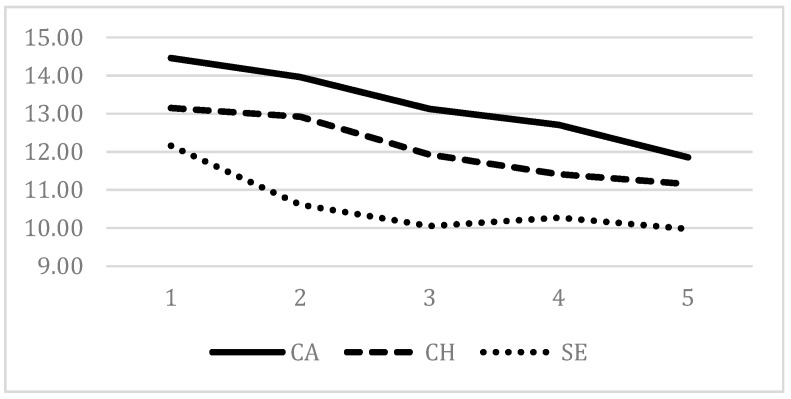
Trends of the decreasing of the level of satisfaction over the five classes. Notes. Satisfaction elements: CA = usefulness for future career; CH = appropriateness of choice; SE = quality of school services;

For services, just the mean difference between the 1^st^ and 2^nd^ class was significant, describing a strong decreasing in the satisfaction between the first two years and a stable tendency for the following years. For choice and career usefulness, each year was not different from the nearest ones, but it was for those most distant, describing a moderate but regular decreasing of the satisfaction over the five classes. The trends of the decreasing are represented in Figure 2.

## 5. Discussion

The study aimed to verify the psychometric properties of the H-Sat Scale, a new scale to measure school satisfaction at high school level, adapted from the CSS [3], a validated scale that measures college satisfaction in a multidimensional perspective. 

Our findings showed good psychometric properties, in terms of internal consistency and validity of the H-Sat Scale, both in the pilot study and in the main one. CFA showed an overlapping structure with the CSS, with acceptable fit indexes, confirming the construct validity of the scale; the significant correlations with the measures used to test concurrent validity (My life as a student and Satisfaction With Life Scale) allow to confirm the adequacy of the external validity. In particular, the correlations between some area of H-Sat and My life as student dimensions seems very consistent, especially in the areas of Relationship (Classmates’ relationships of My life as Student); Study (Praise received for My life as Student); Career usefulness, Choice, Study and Services (School Experience of My life as Student). Furthermore, the high correlation between domain-specific satisfaction and overall life satisfaction is consistent with the socio-cognitive vision of well-being, which sustains a direct relation between the part and the whole [65], and is in line with previous studies [69], confirming the strong relationship between domain-specific satisfaction in a valued context and general life satisfaction.

The relationships between the satisfaction areas appropriateness of choice, effectiveness of his/her study habits and usefulness for future career and the school performance can be confirmed by previous studies that connected GPA, level of achievement and domain-specific satisfaction [88]. Our results show a lower level of satisfaction in the area of choice, utility and services advancing in the school career. Although this is not a longitudinal study, this result could be explained by the fact that in the initial “idealised” view of the school, students would better perceive the limits of the school attended by progressing in their school path.

Moreover, the H-Sat Scale structure covers the dimensions of Konu and Rimpela School Well-being Model, particularly the areas of school conditions (having) and social relationships (loving), confirming that feeling peer support and perceive positive school environment can facilitate educational progress and positive adolescence development and academic adjustement [22,23,24,25,26,27,28,29,30,31,32,33,34,35,36]. In comparison with the previous scales, the H-Sat Scale assesses the concept of school satisfaction as a multidimensional construct with different dimensions, using a reduced number of items despite the variety of information obtained. Compared with previous scales, more focused on general adolescent’s quality of life, our scale focalizes the crucial role of school influence on adolescent’s development and well-being [11,66,73,82].

The novelty of the career areas in the evaluation of school satisfaction leads us to make some additional reflections on its possible uses and the implication for career interventions. H-Sat Scale could be useful to administer in academic context in order to assess satisfaction with school in relation to (1) non intellective variables influencing academic success and performance, such as motivation, self-esteem, self regulation, effort, time management, etc.; (2) positive variables such as resilience, hope, optimism, courage and career adaptability demonstrating to affect the positive attitude toward the future in youth development and in career path; (3) the variables involved in assesment of the effectiveness of school career intervention; (4) to set futher study to corroborate socio-cognitive well-being model as suggest by Lent and Brown underlining the need of empirical evidence in this field (f.e. measuring academic self-efficacy in relation to academic satisfaction) [25,27,28,72,89,90,91]. Lent and Brown underlined also that vocational guidance psychology has been more interested in studying how to support career choice than to focus on the contextual people’s adjustment and satisfaction. For this reason, an evaluation of domain specific satisfaction can lead career intervention, in particular career counseling, to recover its core mission, that is to set practices helping people to improve their well-being and to live more highly functioning lives, rather than simply support their career choice [72].

## 6. Conclusions

The aim of our research was to establish a reliable, multidimensional and domain-specific measure of students’ school satisfaction and to prove its relations with general life satisfaction. Considering the lack of studies on students’ satisfaction with school, we decided to adapt the College Satisfaction Scale to assess contextual satisfaction in adolescents with a short questionnaire useful during the administrations in a battery of tests. We confirmed the previous five-dimensional structure of school satisfaction also in high school students’ sample: appropriateness of choice (CH), quality of school services (SE), relationships with classmates (RE), effectiveness of study habits (ST) and usefulness for future career (CA). The questionnaire consists of 20 items; it showed good psychometric features and confirmed its validity in relations with life satisfaction and quality of life of high school students. The H-Sat Scale can be a valid instrument to help students to identify one or more potential areas of dissatisfaction during the school adjustment. Indeed, professionals and school counsellors can administer this instrument starting from first high school year and set interventions also to promote student persistence and to prevent dropout phenomenon related to ill-being at school. For example, the early evaluation of appropriateness of school chosen and the perceived utility of school in career path could help career counsellor to support students to explore more deeply the meaning of schools in their life trajectories. Moreover, researchers, practitioners and tutoring services through using the H-Sat Scale can better understand the role of each dimension in improving students’ satisfaction or the identification of the dissatisfaction domains that could interfere with the school progress. Indeed, analysing the study habits satisfaction areas students could help to understand which factors foster or impede their academic achievement. The Relational subscale could be useful (1) to set social or community intervention in the classrooms to increase social well-being (2) to help individual students with dissatisfaction levels in this area (i.e., school counselling). Considering the subscale about services, educational administrators can image and set contextual changes in order to improve the quality of the services and school well-being using scientific analysis of students’ opinions. Given the centrality of school for the students’ identity, create a positive academic adjustment based on scientific knowledge of weakness and strengths in satisfactions’ areas could also increase overall life satisfaction, happiness and perceived well-being in adolescence, already starting from the first school year and for the entire duration of the school path. Although our study did not use a longitudinal methodology, school counsellors could support students to face the probable decrease of satisfaction in the area of choice, utility and services advancing in school career. Future longitudinal studies could aim to confirm these results.

### Limitations and Future Studies

The findings of the study should be read in light of its limitations. Firstly, the research was conducted on samples that were not representative of the high school population or completely comparable since we used a convenience sample method. In future studies, the psychometric properties of the H-Sat Scale should be tested with more numerous and more heterogeneous samples at least for sex and types of high school attended. Moreover, since the cross-sectional method did not allow us to know the possible changes of subscales over time and their predictive validity, longitudinal studies should be implemented. The H-Sat Scale is a self-report measure that increases the risk factors associated to the biases of this method, for example related to personality variables.

Future studies could use H-Sat Scale in different nations in order to define if cultural differences exist regarding the structural dimensions of school satisfaction and to confirm/disconfirm psychometrics proprieties of the instrument. The H-Sat Scale has to prove in future its validity using an additional criterion that evaluates psychological variables demonstrating to affect domain-specific satisfaction, such as variables from a positive psychology framework (like hope, optimism, resilience, courage) or in socio-cognitive well-being perspective such as self-efficacy [30]. Future studies should consider these variables in order to verify the existence of stronger relations with life satisfaction. In conclusion, the validation of the instrument on children (8–13 years) samples could extend the utility of the instrument to set interventions in order to improve students’ well-being at all educational levels.

## Figures and Tables

**Table 1:** Exploratory Factor Analysis.

	CA	RE	ST	SE	CH
Item 5	**0.874**	0.017	0.018	−0.026	0.005
Item 10	**0.864**	−0.013	−0.018	−0.066	−0.116
Item 15	**0.846**	−0.010	0.036	0.038	0.030
Item 20	**0.808**	0.029	−0.026	0.077	−0.014
Item 3	−0.023	**0.903**	0.008	−0.050	−0.013
Item 18	−0.031	**0.896**	−0.040	−0.045	−0.058
Item 13	0.059	**0.783**	−0.035	0.073	0.078
Item 8	−0.001	**0.764**	0.077	0.035	−0.016
Item 4	0.051	−0.022	**0.915**	0.015	0.136
Item 19	−0.085	0.063	**0.823**	−0.020	−0.098
Item 14	0.097	0.006	**0.772**	0.003	0.036
Item 9	−0.029	−0.002	**0.700**	0.021	−0.206
Item 7	−0.043	−0.004	0.036	**0.862**	0.039
Item 12	0.017	−0.015	−0.045	**0.798**	0.016
Item 2	0.018	0.011	−0.058	**0.630**	−0.154
Item 17	0.096	0.114	0.141	**0.454**	−0.011
Item 1	0.031	0.053	0.010	0.104	**−0.759**
Item 11	0.165	0.058	0.033	−0.007	**−0.748**
Item 16	0.160	0.029	0.119	0.184	**−0.503**
Item 6	0.168	−0.007	**0.304**	0.072	**−0.393**

*Notes*: Satisfaction elements: CA = usefulness for future career; RE = relationships with classmates; ST = effectiveness of his/her study habits; SE = quality of school services; CH = appropriateness of choice. Weights higher than 0.300 are in bold.

**Table 2:** Scales’ psychometrics.

Scales	Mean	SD	Skewness	Kurtosis
CH	12.27	3.269	−0.125	−0.006
SE	10.75	2.665	−0.097	0.103
RE	13.42	3.444	−0.417	0.002
ST	11.32	3.050	−0.095	0.005
CA	13.38	3.571	−0.163	−0.196

*Notes*: Satisfaction elements: CH = appropriateness of choice; SE = quality of school services; RE = relationships with classmates; ST = effectiveness of his/her study habits; CA = usefulness for future career.

**Table 3:** Reliability: Cronbach’s alpha indexes.

Scales	**Total Sample**	**Linguistic Lyceum**	**Scientific Lyceum**	**Technical Institute**
CH	0.866	0.895	0.827	0.860
SE	0.761	0.768	0.778	0.745
RE	0.880	0.912	0.879	0.859
ST	0.873	0.902	0.880	0.847
CA	0.922	0.927	0.929	0.913

*Notes*: Satisfaction elements: CH = appropriateness of choice; SE = quality of school services; RE = relationships with classmates; ST = effectiveness of his/her study habits; CA = usefulness for future career.

**Table 4:** Concurrent Validity: Correlations between H-Sat and MLS (My Life as a Student) subscales.

Scales	MLS School Experience	MLS Autonomy on Life Decisions	MLS Classmates’ Relationships	MLS Current Life Conditions	MLS Relationships with Family	MLS Praise Received	MLS Help Availability
CH	**0.757** ***	0.113 **	0.235 ***	0.260 ***	0.210 ***	0.432 ***	0.217 ***
SE	**0.525** ***	0.079 *	0.194 ***	0.125 ***	0.177 ***	0.240 ***	0.153 ***
RE	0.247 ***	0.269 ***	**0.727** ***	0.146 ***	0.239 ***	0.268 ***	0.361 ***
ST	**0.519** ***	0.257 ***	0.270 ***	0.454 ***	0.370 ***	**0.755** ***	0.308 ***
CA	**0.777** ***	0.084 *	0.177 ***	0.209 ***	0.198 ***	0.383 ***	0.187 ***

*Notes*: * *p* < 0.05; ** *p* < 0.01; *** *p* < 0.001; Correlations higher than 0.500 are in bold; Satisfaction elements: CH = appropriateness of choice; SE = quality of school services; RE = relationships with classmates; ST = effectiveness of his/her study habits; CA = usefulness for future career.

**Table 5:** Concurrent Validity: Correlations between H-Sat and SWLS and School Performance.

**Scales**	General Life Satisfaction	**School Performance**
CH	0.340 ***	0.246 ***
SE	0.260 ***	−0.002
RE	0.266 ***	0.059
ST	0.454 ***	0.474 ***
CA	0.290 ***	0.145 ***

*Notes*: *** *p* < 0.001; Satisfaction elements: CH = appropriateness of choice; SE = quality of school services; RE = relationships with classmates; ST = effectiveness of his/her study habits; CA = usefulness for future career.

**Table 6:** ANOVA of H-Sat scales for the five classes attended.

Scales	**F**	**d.f.**	***p***
CH	12.633	4	0.000
SE	23.198	4	0.000
RE	1.822	4	0.123
ST	2.032	4	0.088
CA	14.047	4	0.000

*Notes*: Satisfaction elements: CH = appropriateness of choice; SE = quality of school services; RE = relationships with classmates; ST = effectiveness of his/her study habits; CA = usefulness for future career.

## Appendix A

**High School Satisfaction Scale (H-Sat)**

Below you will find a series of statements about how satisfied you are with your school experience. Read each of them carefully and indicate how true they are for you using a scale which goes from “1–Not at all” to “5–Extremely”.

	**I Am Satisfied...**	**Not at all**	**Slightly**	**Moderately**	**Very**	**Extremely**
1	For taking this school *Per aver scelto questa scuola*	1	2	3	4	5
2	Because the classrooms where we carry out our lessons are comfortable *Delle aule in cui vengono svolte le lezioni*	1	2	3	4	5
3	Of the relationships with my classmates *Delle relazioni con i/le miei/mie compagni/e di classe*	1	2	3	4	5
4	About my way of studying *Del mio modo di studiare*	1	2	3	4	5
5	Because my studies will be useful for my educational and/or professional future *Perché penso che questo corso di studi sarà utile per il mio futuro formativo e/o professionale*	1	2	3	4	5
6	Because I like what I am studying in this school *Del fatto che mi piace ciò che studio*	1	2	3	4	5
7	Of the school’s equipment *Delle attrezzature di cui è dotata la mia scuola*	1	2	3	4	5
8	Because I can study well with my classmates *Perché ho dei/delle compagni/e di classe con cui mi trovo bene a studiare*	1	2	3	4	5
9	Of the school goals I am achieving *Degli obiettivi scolastici che sto raggiungendo*	1	2	3	4	5
10	Because I feel that my studies will be useful for my educational and/or professional future career *Perché penso che questo corso di studi avrà un effetto positivo sulla mia futura carriera formativa e/o professionale*	1	2	3	4	5
11	For having undertaken this school *Per aver intrapreso questo corso di studi*	1	2	3	4	5
12	Of the services for the students (secretariat, library, gym, etc..) *Dei servizi per noi studenti/esse (segreteria, biblioteca, palestra, etc.)*	1	2	3	4	5
13	Because I can count on the help of my classmates *Perché posso contare sull’aiuto dei miei compagni di classe*	1	2	3	4	5
14	For my motivation in the study *Della mia motivazione nello studio*	1	2	3	4	5
15	Because this school will have a positive effect on my future professional career *Perché in questa scuola sto mettendo le basi per la mia carriera lavorativa*	1	2	3	4	5
16	Because, after all, this school course suits me *Perché, tutto sommato, questa scuola sembra fatta su misura per me*	1	2	3	4	5
17	Of the availability of those who work in the school toward the students *Della disponibilità di chi lavora nella scuola verso gli/le studenti/esse*	1	2	3	4	5
18	About my friendship with my classmates *Dei buoni rapporti di amicizia con i/le miei/mie compagni/e di classe*	1	2	3	4	5
19	For my school results *Dei miei risultati scolastici*	1	2	3	4	5
20	Because what I’m learning in this school will be useful to find a good job *Perché frequentare questa scuola mi sarà utile per trovare una futura occupazione lavorativa*	1	2	3	4	5
